# Global glomerulosclerosis proportions predict nephropathy progression in IgA nephropathy: a multicenter retrospective analysis with propensity score matching

**DOI:** 10.1080/0886022X.2025.2486567

**Published:** 2025-05-05

**Authors:** Wenli Zou, Yueming Liu, Baihui Xu, Juan Wu, Wei Shen, Wei Zhang, Jiawei Zhang, Yao Meng, Yan Zhu, Jingting Yu, Jianguang Gong, Xiaogang Shen, Bin Zhu

**Affiliations:** a Urology & nephrology Center, Department of nephrology, Zhejiang Provincial People’s Hospital, affiliated People’s Hospital, Hangzhou Medical College, Hangzhou, Zhejiang, China; b Department of nephrology, Hangzhou Hospital of Traditional Chinese Medicine affiliated to Zhejiang Chinese Medical University, Hangzhou, Zhejiang, China

**Keywords:** Global glomerulosclerosis, IgA nephropathy, kidney biopsy, kidney failure, propensity score matching

## Abstract

**Background:**

Immunoglobulin A nephropathy (IgAN) is the most common primary glomerular disease worldwide. The role of global glomerulosclerosis (GS) in the patients with IgAN remains controversial. The study aims to evaluate the effect of GS on the kidney outcome in the patients with IgAN.

**Methods:**

Based on the median of GS proportion, patients were divided into two groups (GS1 and GS2). Thereafter, the clinical, demographic, and treatment characteristics were evaluated by propensity score matching, Kaplan-Meier survival curves, Cox regression analyses. Next, the receiver operating characteristic curve analysis, continuous net reclassification improvement and integrated discrimination improvement were constructed to assess whether the model contained GS proportion could refine risk prediction and clinical utility.

**Results:**

In this three-center retrospective study, a total of 1,626 IgAN patients were recruited. Both in the full and matched cohort, higher GS proportions were found to be independent prognostic factors for the kidney survival *via* Kaplan-Meier analysis (*p* < 0.05, *p* < 0.05, respectively). Additionally, the multivariate Cox regression models identified higher proteinuria, decreased eGFR, higher score of tubular atrophy/interstitial fibrosis (T), higher GS proportions as independent prognostic factors for poor kidney outcomes, and corticosteroids therapy was a protective indicator of kidney outcomes. Lastly, the prediction model based on these prognostic factors were validated to be accurately predict the kidney outcome when including GS proportions.

**Conclusions:**

GS proportions are independent predictors for poor prognosis in IgAN patients.

## GRAPHICAL ABSTRACT

**Figure:**
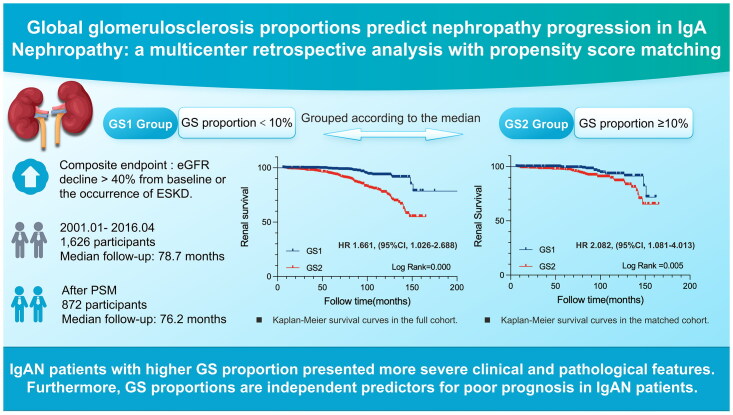

## Introduction

IgAN is the most prevalent primary glomerulonephritis worldwide, with an overall incidence of approximately 2.5 per 100,000 individuals [1], and it is the dominant cause of kidney failure in East Asian countries. Given the poor outcomes and various clinical courses associated with IgAN, to explore the factors associated with the prognosis is extremely important.

Renal histological evaluation is essential for the prediction of IgAN outcome. The Oxford classification, which includes evaluation of mesangial hypercellularity (M), endocapillary hypercellularity (E), segmental glomerulosclerosis (S), tubular atrophy/interstitial fibrosis (T), and crescents (C), referred to as the MEST-C score, was developed to predict the outcomes of IgAN [2–4]. This scoring system is highly reproducible and has been extensively validated [5,6]. Moreover, global glomerulosclerosis (GS), a chronic pathological change, has been defined as a scarring lesion or hyaline deposition involving all of a glomerular tuft. GS is excluded in the original Oxford classification because of its significant correlation with tubular atrophy/interstitial fibrosis [7]. However, the original Oxford study included a limited sample size and excluded patients with severe IgAN. Furthermore, recent reports have indicated that GS is a strong predictor of long-term kidney outcomes independent of T scores in patients with IgAN [8,9]. Consequently, we aimed to perform a retrospective, three-center cohort study with a larger sample size, and a longer follow-up was performed to examine the relationship between GS and kidney outcomes in IgAN patients.

## Materials and methods

### Study population

This multicenter retrospective study included patients with IgAN, with diagnoses confirmed through biopsy, admitted at Zhejiang Provincial People’s Hospital, Hangzhou Hospital of Traditional Chinese Medicine, and Qinghai Provincial People’s Hospital from January 2001 to April 2016. The inclusion criteria were as follows: (1) kidney biopsy-proven primary IgAN; (2) age ≥ 14 years; and (3) follow-up duration > 6 months. The exclusion criteria were as follows: (1) fewer than eight glomeruli in the biopsy renal tissue section of one patient; (2) follow-up duration < 6 months; (3) initial estimated glomerular filtration rate (eGFR) < 15 mL/min/1.73 m^2^; (4) detection of secondary causes of IgAN such as liver or inflammatory bowel diseases, other autoimmune disorders, infections, and Henoch-Schönlein purpura; (5) detection of inherited kidney disease.

This study was conducted in accordance with the principles embodied in the Declaration of Helsinki.

### Data collection

Demographic and clinical data, including age; sex; 24-h urine total protein (UTP); serum creatine; serum immunoglobulin and complement levels; MEST-C score, and medications, were retrospectively collected at the time of renal biopsy. Serum creatinine levels at follow-up were retrospectively collected. The eGFR was calculated using the Chronic Kidney Disease Epidemiology Collaboration (CKD-EPI) equation.

The kidney biopsy was performed for light microscopy and direct immunofluorescence. Tissues for histological analysis were either fixed in 10% formalin and embedded in paraffin or fixed in 3% paraformaldehyde and embedded in polycol methacrylate. Paraffin-embedded sections were stained with hematoxylin and eosin, periodic acid-Schiff, periodic acid-silver methenamine, and trichrome. Snap-frozen renal tissues were adopted for the immunofluorescence analysis. Six-micron slides were stained with fluorescein-conjugated antibodies specific to human IgG, IgM, IgA, C3, and C4. The degree of immunofluorescence was scored on a scale of 0 to 4 (negative, +, ++, +++, and ++++). Histological sections were reviewed by two experienced renal pathologists who were blinded to the clinical data. Biopsies containing fewer than eight glomeruli were deemed inadequate for scoring and excluded from the analysis.

GS was defined as a scarring lesion or hyaline deposition involving all of a glomerular tuft. For statistical analysis, when treating the percentage of GS as a continuous variable, the model estimate is unstable when GS value is high due to limited number and skewed distribution of GS (shown in the supplementary figure). Then eligible patients were divided into the GS1 group (0–10% of glomeruli with GS) and the GS2 (≥10% of glomeruli with GS) group based on the median value of GS proportions.

### Definition of outcomes

The composite endpoint was defined as eGFR decline > 40% from baseline or the occurrence of end-stage kidney disease (ESKD) (defined as eGFR < 15 mL/min/1.73 m^2^ and the need for dialysis or kidney transplantation).

### Statistical analyses

Continuous variables were expressed as the mean and standard deviation for normally distributed data or as the median and interquartile range for non-normally distributed data. Differences in means between groups were compared using Student’s t-test or Kruskal-Wallis H tests based on their distributions. Categorical variables were presented as frequencies and percentages. Differences in percentages between groups were compared using χ^2^ or Fisher’s exact tests. Immunoglobulin and complement levels were categorized into the high and low groups based on the median levels. A p value of < 0.05 was considered statistically significant. Concerning the imbalance of the covariates at baseline in the full cohort, we then adopted the propensity score matching (PSM) (1:1) for the further analyses with a caliper requirement of 0.02. After all propensity score matches were performed, the balance of baseline covariates was subsequently evaluated. A p value of >0.05 indicated a negligible difference between the groups.

Survival curves of the composite kidney endpoints between groups were summarized and compared using the Kaplan-Meier method with the log-rank test. Cox regression models were adjusted for relevant covariates. To identify the independent risk factors, univariate Cox regression models were used, and baseline variables considered to be clinically relevant or with a *p* < 0.2 in the univariate analysis were entered into a multivariate Cox proportional hazards regression model using the ‘forward LR’ method. Hazard ratios (HRs) and 95% confidence intervals (CIs) were calculated.

In the original Oxford classification, GS was excluded due to its significant correlation with interstitial fibrosis (*r* = 0.8) and tubular atrophy (*r* = 0.7). Subgroup and interaction analyses were performed using Cox proportional hazards models adjusted for all covariates (except the variable of the subgroup of concern) to examine the relationship between the T score and GS.

In both the full and matched cohorts, the prediction accuracy of the model with or without GS was assessed with receiver operating characteristic (ROC) curves using the ‘pROC’ R package, and areas under the curve (AUCs) were established. Reclassification was assessed *via* continuous net reclassification improvement (cNRI) and integrated discrimination improvement (IDI) determined the R package. Both cNRI and IDI were adapted for censoring the assessment of the model with or without adding GS to the baseline model; other existing risk factors were derived from the multivariable-adjusted Cox regression model. Values significantly greater than zero indicated an improvement in discrimination. All statistical analyses were conducted using the SPSS software version 25.0 (IBM Corp., Armonk, NY, USA).

## Results

A total of 1,626 patients were included in our study. Based on the median of 10% of GS proportions, the patients were divided into the two groups: the GS1 group (GS proportion <10%) with 817 (50.2%) patients and the GS2 group (GS proportion ≥10%) with 809 (49.4%) patients. After PSM, 436 patients in the GS1 group were successfully matched to 436 patients in the GS2 group (Table 1).

**Table 1.** Baseline demographic and clinical characteristics of IgAN patients.

Characteristic	Before matching			after matching (1:1)		
	GS1	GS2	*p*-value	GS1	GS2	*p*-value
Participants	817 (50.2%)	809 (49.4%)		436 (50%)	436 (50%)	
Clinical characteristics at biopsy						
Age, years	30 ± 15	36 ± 14	<0.001	33 ± 15	34 ± 13	0.07
Male (n,%)	342 (41.9%)	363 (44.9%)	0.230	183 (42.0%)	179 (41.1%)	0.418
Clinical features						
Scr	65.0 ± 26.9	85.0 ± 41.1	<0.001	72.0 ± 29.0	73.7 ± 29.8	0.544
24-h UTP > 1g	221 (27.1%)	357 (44.1%)	<0.001	157 (36.0%)	145 (33.3%)	0.434
eGFR, ml/min per 1.73m^2^	114.9 ± 28.7	89.1 ± 44.2	<0.001	102.8 ± 33.3	103.2 ± 30.8	0.378
Serum IgA, g/L	2.82 ± 1.42	3.01 ± 1.41	0.035	2.91 ± 1.47	2.92 ± 1.36	0.753
Serum IgM, g/L	1.37 ± 0.84	1.28 ± 0.90	0.016	1.38 ± 0.89	1.36 ± 0.94	0.711
Serum IgG, g/L	10.80 ± 3.57	11.00 ± 3.77	0.603	10.80 ± 3.48	10.90 ± 3.49	0.817
C3, g/L	0.91 ± 0.31	0.89 ± 0.30	0.911	0.91 ± 0.32	0.91 ± 0.29	0.998
C4, g/L	0.20 ± 0.09	0.21 ± 0.09	<0.001	0.21 ± 0.09	0.21 ± 0.08	0.714
MEST-C score						
M1 (%)	433 (53.0%)	503 (62.2%)	<0.001	266 (61.0%)	253 (58.0%)	0.408
E1 (%)	54 (6.6%)	38 (4.7%)	0.107	32 (7.3%)	24 (5.5%)	0.334
S1 (%)	265 (32.4%)	510 (63.0%)	<0.001	231 (53.0%)	204 (46.8%)	0.078
T1 + T2 (%)	49 (6.0%)	296 (36.6%)	<0.001	49/0 (11.2%, 0.0%)	53/1 (12.2%/0.2%)	0.552
C1 + C2 (%)	385 (47.1%)	419 (51.8%)	0.06	244/1 (56.0%/0.2%)	220/1 (50.5%/0.2%)	0.264
Medications, (n,%)						
RAS blockade	609 (74.5%)	666 (82.3%)	<0.001	334 (76.6%)	349 (80.0%)	0.250
Corticosteroids	448 (54.8%)	581 (71.8%)	<0.001	300 (68.8%)	287 (65.8%)	0.386

GS: global glomerulosclerosis; Scr: serum creatinine; 24-h UTP: 24-h urinary total protein; eGFR: estimated glomerular filtration rate; C3: serum complement 3; C4: serum complement 4; M: mesangial hypercellularity; E: endocapillary hypercellularity; S: segmental glomerulosclerosis; T: interstitial fibrosis and tubular atrophy; C: crescents; RAS blockade: renin-angiotensin system blockade.

### Baseline characteristics of the participants

Compared to patients in the GS1 group, patients in the GS2 group were more likely to be older (36 vs. 30 years, *p* < 0.001) and with a lower eGFR (89.1 vs. 114.9, *p* < 0.001). The GS2 group had a higher percentage of patients with 24-h UTP >1 g than the GS1 group (44.1% vs. 27.1%, *p* < 0.001). The percentage of patients who received renin-angiotensin system blockade and corticosteroid therapies was lower in the GS1 group than that in the GS2 group (*p* < 0.001). The percentages of M1 (62.2% vs. 53.0%, *p* < 0.001), S1 (63.0% vs. 32.4%, *p* < 0.001), and T1 + T2 (36.6% vs. 6.0%, *p* < 0.001) were higher in the GS2 group compared with those in the GS1 group.

The baseline clinical characteristics of the GS1 and GS2 groups were matched using the PSM method. A total of 872 patients (436 in the GS1 group and 436 patients in the GS2 groups) were included in the study after the PSM analysis. In the propensity score-matched cohort, no statistically significant differences in baseline covariates at baseline were observed between the two groups (Table 1).

### Relationship between GS proportions and renal survival in the full cohort

**Figure 1.**
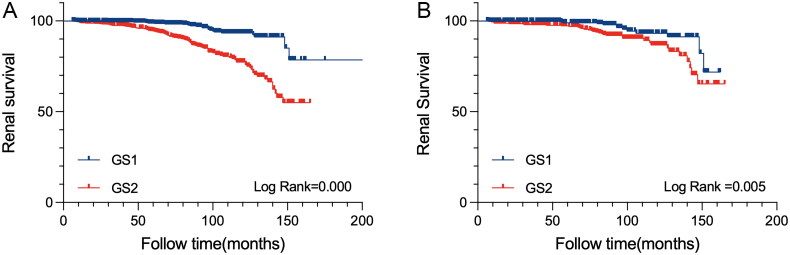
A Kaplan-Meier survival curve for progression to the kidney failure outcome. Kidney failure was defined as 40% decrease of the eGFR from baseline, kidney replacement therapy including dialysis or kidney transplantation. (A) and (B) showed Kaplan-Meier survival curves for the comparisons among different levels of global glomerulosclerosis proportions in the full cohort and the matched cohort, respectively.

Of the 1,626 patients, with a median follow-up period of 78.7 months, 27 (3.4%) in the GS1 group and 104 (12.9%) in the GS2 group reached the primary outcome. Kaplan-Meier analysis showed that patients in the GS2 group had a higher risk of developing kidney failure as compared with those in the GS1 group (log-rank *p* < 0.05, Figure 1A).

Cox regression analysis was performed to determine the clinical characteristics that might influence the renal prognosis of patients with IgAN (Table 2). Univariate Cox regression analysis revealed that patients with GS proportions of ≥10% were at an increased risk for developing kidney failure. Multivariate Cox regression analysis indicated that this association remained after adjusting for related covariates (HR for ≥ 10% vs. <10%: 1.661, 95% CI = 1.026–2.688, *p* = 0.039). Other covariates included in the final multivariate Cox regression model were 24-h UTP levels (HR for ≥ 1 g vs. <1 g: 2.745; 95% CI = 1.857–4.058, *p* < 0.001), decreased eGFR (HR for CKD3 vs. CKD1: 3.140, 95% CI = 1.995–4.942, *p* < 0.001; HR for CKD4 vs. CKD1: 6.970, 95% CI = 3.045–15.957, *p* < 0.001), lower serum IgA levels (HR for higher vs. lower levels: 0.645, 95% CI = 0.455–0.915, *p* = 0.014), higher T score (HR for T1 + T2 vs. T0: 2.589, 95% CI = 1.731–3.871, *p* < 0.001), and higher M score (HR for M1 vs. M0: 1.600, 95% CI = 1.092–2.345, *p* = 0.016).

**Table 2.** Univariate and multivariate cox regression analyses of the risk factors in the cohort study and after propensity score matching.

Characteristic	The full Cohort (*n* = 1626)			The matched Cohort (*n* = 872)		
	Univariate analyses	Multivariate analyses		Univariate analyses	Multivariate analyses	
	*p*-value	HR (95%CI)	*p*-value	*p*-value	HR (95%CI)	*p*-value
Age (years)	0.042			0.159		
Gender (males vs. female)	0.133			0.764		
24-h UTP (g)	<0.001	2.745 (1.857, 4.058)	<0.001	<0.001	3.493 (1.869, 6.528)	<0.001
eGFR						
CKD2 VS. CKD1	<0.001	1.426 (0.904, 2.247)	0.127	0.048	1.370 (0.698, 2.689)	0.360
CKD3 VS. CKD1	<0.001	3.140 (1.995, 4.942)	<0.001	<0.001	2.968 (1.365, 6.454)	0.006
CKD4 VS. CKD1	0.023	6.970 (3.045, 15.957)	<0.001	0.002	4.832 (1.102, 21.180)	0.037
Serum IgA	0.141	0.645 (0.455, 0.915)	0.014	0.213		
Serum IgM	0.019			0.646		
Serum IgG	<0.001			0.145		
C3	0.994			0.148		
C4	0.038			0.013		
RAS blockade therapy	0.277			0.874		
Corticosteroids therapy	0.146			0.074	0.454 (0.250, 0.824)	0.009
GS2 vs. GS1	<0.001	1.661 (1.026, 2.688)	0.039	0.007	2.082 (1.081, 4.013)	0.028
MEST-C score						
M	0.001	1.600 (1.092, 2.345)	0.016	0.773		
E	0.379			0.072		
S	<0.001			0.168		
T1 +T2 vs. T0	<0.001	2.589 (1.731, 3.871)	<0.001	<0.001	3.674 (1.945, 6.939)	<0.001
C1+ C2 vs. C0	<0.001			0.706		

24-h UTP: 24-h urinary total protein; eGFR: estimated glomerular filtration rate; CKD: chronic kidney disease; C3: serum complement 3; C4: serum complement 4; RAS blockade: renin-angiotensin system blockade; GS: global glomerulosclerosis; M: mesangial hypercellularity; E: endocapillary hypercellularity; S: segmental glomerulosclerosis; T: interstitial fibrosis and tubular atrophy; C: crescents.

### Association between GS proportions and renal survival in the matched cohort

After PSM, a total of 872 patients with a median follow-up period of 76.2 months were included in the matched cohort. Among them, 13 patients (3.0%) in the GS1 group and 35 patients (8.0%) in the GS2 group achieved the primary outcome. In the matched cohort, Kaplan-Meier analysis showed that patients in the GS2 group had a higher risk of developing kidney failure compared to those in the GS1 group (log-rank *p* < 0.05, Figure 1B). Univariate Cox regression analysis revealed that patients with GS proportions ≥10% were significantly associated with an increased risk of kidney failure. Multivariate Cox regression analysis indicated that this association remained after being adjusted for related covariates (HR for ≥ 10% vs. <10%: 2.082, 95% CI = 1.081–4.013, *p* = 0.028). Other covariates, including 24-h UTP levels (HR for ≥ 1 g vs. <1 g: 3.493, 95% CI = 1.869–6.528, *p* < 0.001), decreased eGFR (HR for CKD3 vs. CKD1: 2.968; 95% CI = 1.365–6.454, *p* = 0.006; HR for CKD4 vs. CKD1: 4.832, 95% CI = 1.102–21.180, *p* = 0.037), and higher T score (HR for T1 + T2 vs. T0: 3.674, 95% CI = 1.945–6.939, *p* < 0.001), remained significantly associated with a high cumulative incidence of kidney failure, which was consistent with the analyses in the full cohort. Additionally, corticosteroid intervention (HR for ‘corticosteroid intervention’ vs. ‘no corticosteroid intervention’: 0.454, 95% CI = 0.250–0.824, *p* = 0.009) was associated with reduced risk of kidney progression in IgAN patients.

### T score subgroup analysis and interaction effect analysis

Concerning the possible correlation between the percentage of glomerular sclerosis and the T scores, we then investigated the relationship between the percentage of glomerular sclerosis and the T score in our cohort. In the full cohort, subgroup analyses indicated that GS severity was more significantly associated with kidney failure in patients with a T0 score (HR for GS2 vs. GS1: 2.131, 95% CI = 1.186–3.380, *p* = 0.011) compared with those with a T1 + T2 score (HR for GS2 vs. GS1: 1.065, 95% CI = 0.368–3.087, *p* = 0.907). Consistently, GS severity was more significantly associated with kidney failure in patients with a T0 score (HR for GS2 vs. GS1: 3.097, 95% CI = 1.355–7.186, *p* = 0.008) compared with in those with a T1 + T2 score (HR for GS2 vs. GS1: 1.753, 95% CI = 0.424–7.244, *p* = 0.438) in the matched cohort (Table 3).

**Table 3.** Subgroup and interaction effects analyses in the full and matched cohort.

	Subgroup		No.of events /total	HR (95%CI)	*p*-value for interaction
Model in the full cohort	TO	GS1	23/768	reference	0.701
		GS2	38/513	2.131 (1.186, 3.830)	
	T1 + T2	GS1	4/49	reference	
		GS2	66/196	1.065 (0.368, 3.087)	
Model in the matched cohort	TO	GS1	9/387	reference	0.512
		GS2	22/382	2.784 (1.266, 6.120)	
	T1 + T2	GS1	4/49	reference	
		GS2	13/54	1.753 (0.424, 7.244)	

A Spearman analysis between GS and T scores in the present study showed a correlation coefficient of 0.455 (*p* < 0.05) in the full cohort and 0.077 (*p* < 0.02) in the matched cohort. The interaction terms of GS with T lesions were not significant in either the full cohort (*p* = 0.701 for the interaction term) or the matched cohort (*p* = 0.512 for the interaction term).

### Assessment of multivariate prediction models with GS proportions

**Figure 2.**
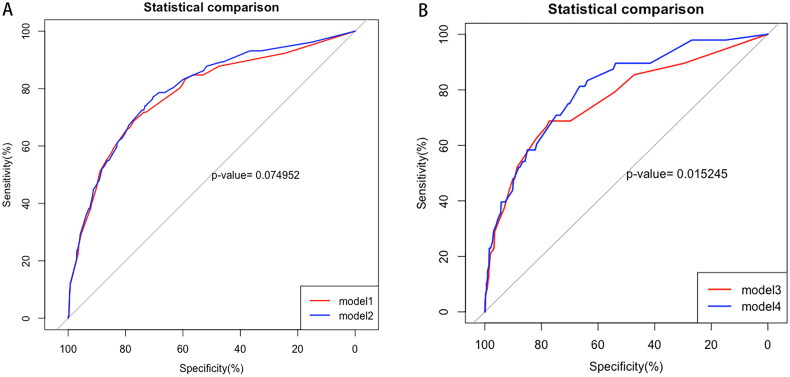
Receiver operating characteristic curves of the kidney failure outcome at 5-year according to the survival model. (A) and (B) showed receiver operating characteristic curves of the survival model in the full cohort and the matched cohort, respectively. The cox survival model 1 and model 3 comprised the statistically significant variables including 24h-UTP, eGFR, corticosteroids use and T score, model 2 and model 4 was adjusted for the above variables plus global glomerulosclerosis proportions.

ROC curves were used to investigate the predictive power of GS proportions with respect to the progression of IgAN. In the full cohort, model 1 (Figure 2(A)) consisted of statistically significant variables from the multivariable-adjusted Cox regression model after PSM, such as 24-h UTP, eGFR, corticosteroid use, and T-score. The AUC was 0.782 (95% CI = 0.7378 – 0.8267) for model 1 and 0.795 (95% CI = 0.753 – 0.837) for model 2 (when the GS proportions were added). In the matched cohort, model 3 (Figure 2(B)) also included 24-h UTP, eGFR, corticosteroid use, and T score; the AUC of model 3 was 0.764 (95% CI = 0.6859 – 0.8427). When the GS proportions were added (model 4), the ROC curves indicated a significantly higher AUC of 0.804 (95% CI = 0.74–0.868, *p* = 0.015) (Figure 2(B)).

The predictive performance of multivariable-adjusted Cox regression models with and without GS proportions was compared. The addition of GS proportions improved discrimination, as assessed by an increase in cNRI and IDI values in both the full and matched cohorts. In the matched cohort, IDI showed a significant increase (*p* = 0.001) (Table 4).

**Table 4.** Prediction performance of the cox model with or without global glomerulosclerosis proportions.

	cNRI (99.9% CI)	IDI (99.9% CI)
Model in the full cohort	Reference	Reference
Plus higher global glomerulosclerosis proportions	0.12 (−0.35, 0.45)	0.002 (−0.003, 0.016)
Model in the matched cohort	Reference	Reference
Plus higher global glomerulosclerosis proportions	0.01 (−0.41, 0.47)	0.031 (0.019, 0.265)

Prediction performance was evaluated using the 5-year risk of a 40% decline in eGFR or ESKD. Significant NRI and IDI values are those whose 99.9% CI do not include zero. CI, confidence interval; cNRI, continuous net reclassification improvement; IDI, integrated discrimination improvement.

## Discussion

The present multicenter study with a large sample size and a longer follow-up period showed that a higher proportions of GS was associated with an increased risk of developing kidney failure in patients with IgAN in both the full cohort and the propensity score-matched cohort. After adding global glomerulosclerosis, the multivariable models showed improved discriminatory power and the prediction accuracy, indicating that a higher proportion of GS may be a predictor for kidney progression in IgAN.

IgAN is a common progressive glomerulonephritis that presents with various clinical features, mainly hematuria and/or proteinuria. The Oxford classification of IgAN has been adopted for the pathological evaluation of renal outcomes [2,7,10] and has been validated by several studies. However, GS is excluded from the Oxford classification owing to its high correlation coefficient with interstitial fibrosis/tubular atrophy. However, the original Oxford study included a small sample size cohort, and excluded severe IgAN patients. An increased proportion of GS can lead to glomerular hypertension and hyperfiltration in remaining nephrons. The adaptive response leads to podocyte hypertrophy and nephron degeneration initially but later promotes kidney disease progression.

Reproducibility and clinical relevance are considered the most important attributes to evaluate histopathological classifications. GS was a candidate predictor of kidney prognosis with relevant reproducibility with an intraclass correlation coefficient of > 0.6 [11,12]. Some cohort studies also identified GS as a major risk factor for IgAN progression [8,13]. Chung et al. suggested that the number of globally sclerotic glomeruli could be an independent risk factor for predicting the renal outcomes in IgAN, especially in those with a T0-score in the Oxford classification [9]. Liu et al. established a nomogram for predicting the 3- and 5-year prognoses of the IgAN patients and suggested that glomerulosclerosis could accurately predict IgAN progression [14]. Chen et al. also included glomerulosclerosis in their prediction model of the Nanjing IgAN Risk Stratification System [15]. In this study, we included a larger sample size of patients and employed multiple statistical methods to explore the role of GS in the kidney progression in IgAN. This study indicated that patients with GS2 have a higher risk of kidney progression compared with those with GS1 in both the full cohort and the matched cohort. We found an improvement in the AUC value and discrimination, as assessed by an increase in cNRI and IDI values, upon adding GS to the model.

The T-score is an important indicator of poor prognosis [16]. Remarkably, our data indicated that the GS2 group had more patients who presented with more severe tubulointerstitial lesions than the GS1 group (36.6% vs. 6.0%). Our multivariate Cox regression analysis also indicated that T1/2 was a significant risk factor for kidney outcomes, with HRs of 2.589 (T1 + 2 vs. T0: 95% CI = 1.731–3.871) in the full cohort and 3.674 (T1 + 2 vs. T0: 95% CI = 1.945–6.939) in the matched cohort. In the Oxford study, the correlation coefficient for IF/TA and GS suggested that these two variables were closely linked [7]. Thus, the Oxford classification includes IF/TA as the T-score rather than a variable of GS. Considering the small sample size and rather strict inclusion criteria in the original Oxford study, we performed Spearman correlation analysis to show that the correlation coefficient between GS proportions and T-scoring (*r* = 0.455, full cohort; *r* = 0.077 in the matched cohort. The interaction terms of GS with T lesions were not significant in either the full cohort (*p* = 0.701) or the matched cohort (*p* = 0.512). Subgroup analysis revealed that a higher GS level was significantly associated with a higher risk of kidney failure in the T0 subgroup. Under certain conditions, the T-score might not be consistent with GS, particularly in patients with medical exposure or a long history of hypertension.

No consensus has been established with respect to the role of corticosteroid intervention in IgAN [17–19]. In the STOP-IgAN trial, immunosuppressive therapy for high-risk IgAN patients did not significantly improve the outcome, and more adverse effects were observed [20,21]. The TESTING study reported potential renal benefits of oral methylprednisolone in high-risk patients with IgAN. However, it was also associated with an increased risk of serious adverse events, primarily with high-dose therapy [22,23]. In our study, patients in the GS2 group were more likely to receive corticosteroid therapy, and the multivariable-adjusted Cox regression model showed that corticosteroid therapy was a protective indicator of kidney outcomes (HR = 0.454, 95% CI = 0.250–0.824, *p* = 0.009). However, the dosage and course of corticosteroid therapy, as well as treatment-related adverse events, were not monitored in our study. Hence, whether corticosteroid therapy improves kidney outcomes needs to be confirmed in larger, reasonably designed clinical studies.

This present study has some limitations. First, this was a retrospective study and was subject to the inherent biases. Second, the dosage and course of corticosteroids, RAS blockade, as well as the other immunosuppressive therapies were not obtained, we could not have a deeper discussion about the role of drugs on the kidney progression in IgAN. Furthermore, our findings need validation in other cohorts, hence, more multicenter studies with a large sample size and a longer follow-up are in demand in the future.

## Conclusion

IgAN patients with higher global glomerulosclerosis proportion presented more severe clinical and pathological features. Furthermore, a global glomerulosclerosis proportion above 10% in the glomeruli indicates a poor prognosis for patients with IgAN.

## Supplementary Material

Supplementary figure 1.tiff

Supplementary figure 2.tiff

## Data Availability

All data generated or analyzed during this study are included in this article. Further enquiries can be directed to the corresponding author.
