# Supplementary figures and images for: Global glomerulosclerosis proportions predict nephropathy progression in IgA nephropathy: a multicenter retrospective analysis with propensity score matching
Source: Ren Fail. 2025 May 5;47(1):2486567. doi: 10.1080/0886022X.2025.2486567 (PMC12057779; doi:10.1080/0886022X.2025.2486567)

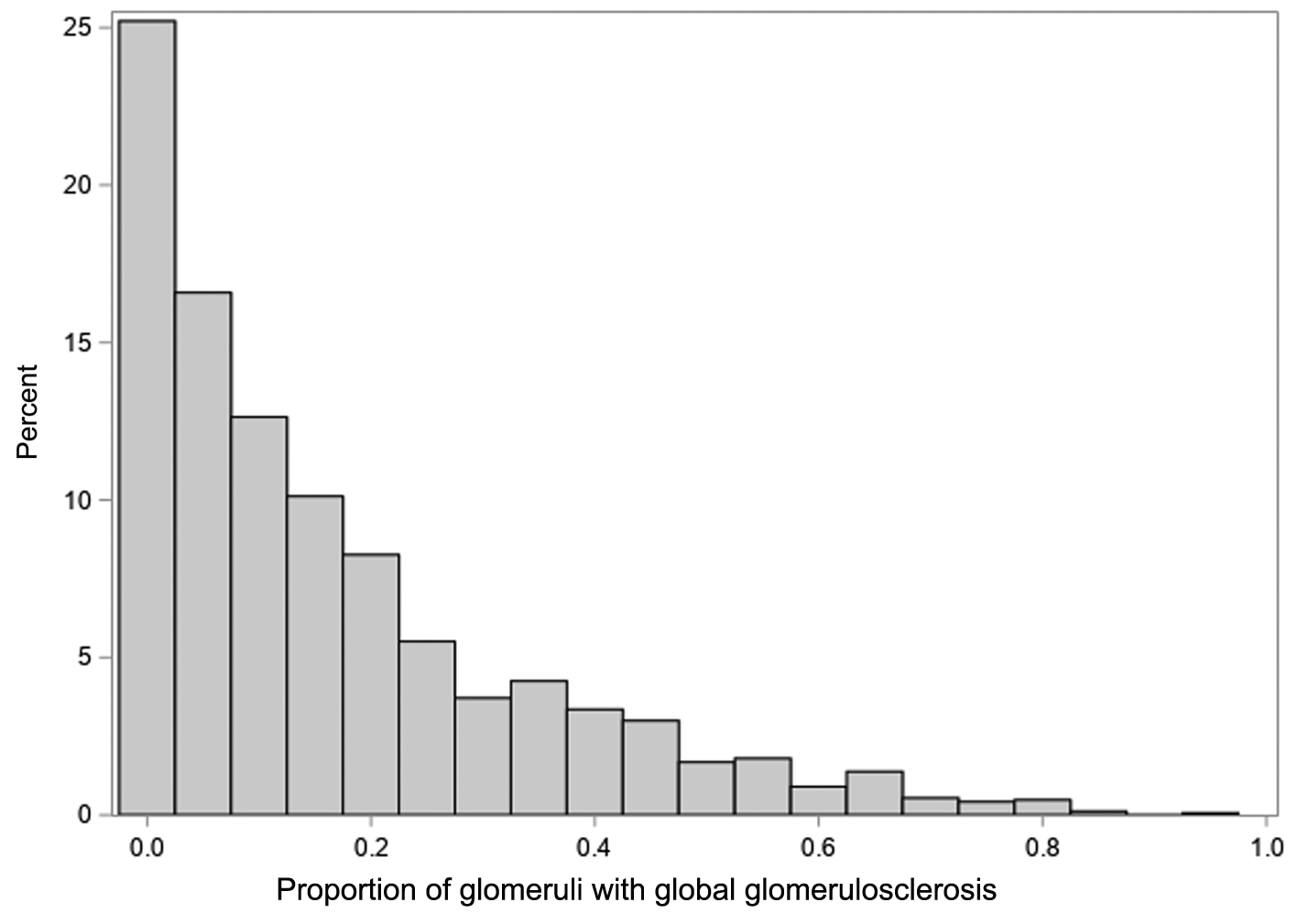

Supplement: Supplementary figure 1.tiff [file IRNF_A_2486567_SM0556.tiff]

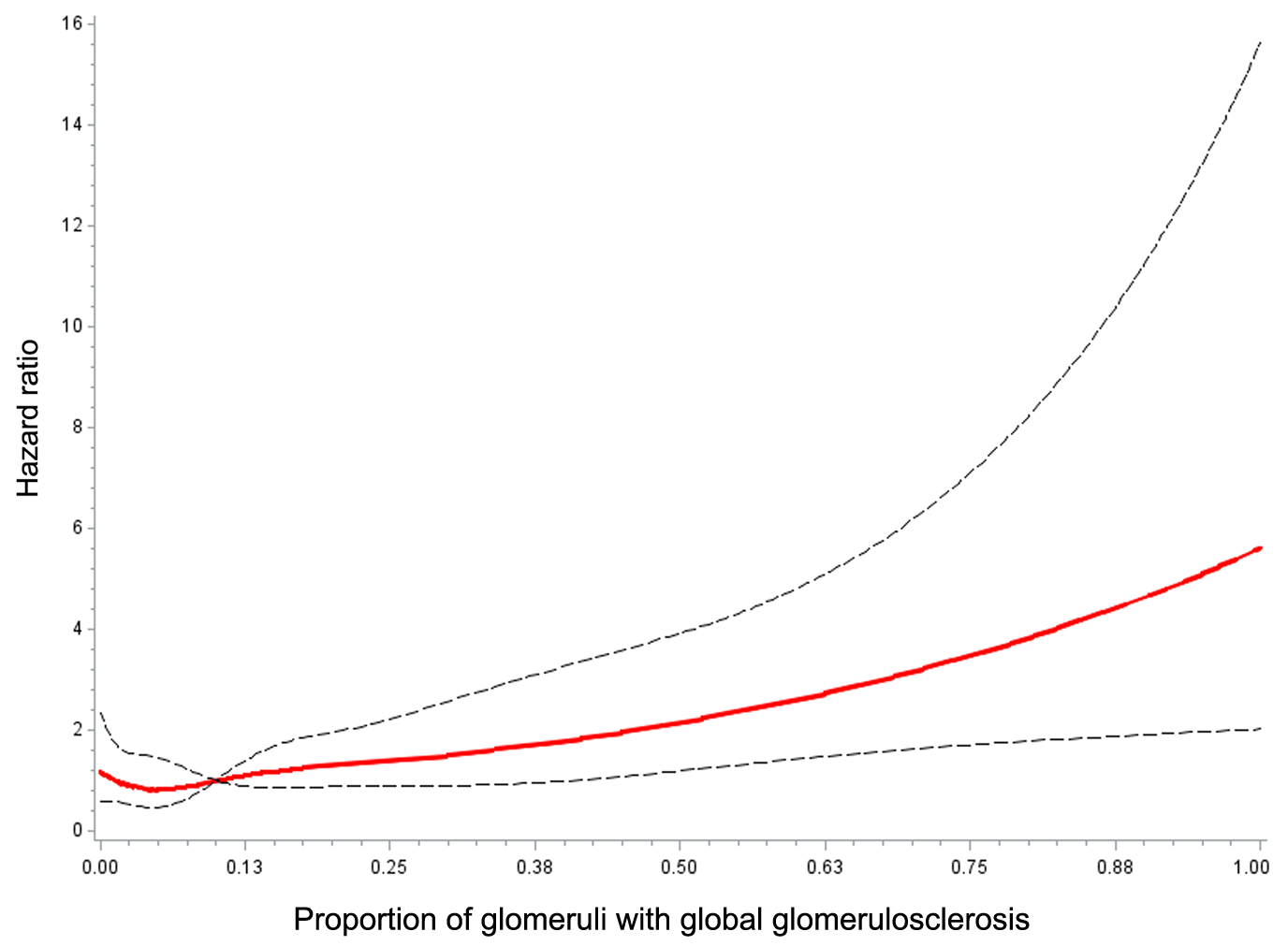

Supplement: Supplementary figure 2.tiff [file IRNF_A_2486567_SM0555.tiff]
